# The Hospital Chapel

**Published:** 1908-03-14

**Authors:** G. Arbour Stephens


Sir,—It ill becomes the Editor of what is supposed to be a non-political journal to make sneering remarks at the Free Churches as you do in your article on the Newcastle chapel. There is no fear of the *morale* of the institution suffering at the hands of the Evangelical Churches.

Had I known you intended casting aspersions in such a way I would not have subscribed to your paper.

I may also remark that I did expect you to have the courtesy to acknowledge the receipt of my monograph on Calcium Permanganate; but as it is written by a Nonconformist sectarian it may affect the *morale* of your paper to refer to it.

Yours truly,
G. Arbour Stephens.

∴ Dr. Stephens can scarcely have read our article carefully, or he would not have fallen into the error which his letter displays. It contains no sneering remarks, as he must do us the justice to admit if he re-reads it, nor are any aspersions cast upon any religious body. Our point was and is that it is unthinkable that in this country any body of Governors of a great general hospital should deliberately and finally determine to deprive its in-patients of all spiritual ministrations and opportunities for worship during the period of their illness. That is why in our judgment the Newcastle decision should be promptly reversed, as we hope it may be, and a special meeting of the Governors called for that purpose. In reply to Dr. Stephens' second complaint, we have not space to acknowledge the receipt of every monograph that is sent to us.—Ed. The Hospital.

